# Low estimated glomerular filtration rate is an independent risk factor for higher hydroxychloroquine concentration

**DOI:** 10.1007/s10067-023-06576-x

**Published:** 2023-03-20

**Authors:** Xue Zhong, Yue-bo Jin, Qin Zhang, Si-lu Liu, Jing He

**Affiliations:** 1 Department of Pharmacy, Peking University People’s Hospital, Beijing, China; 2 Department of Rheumatology & Immunology, Peking University People’s Hospital, No. 11 South Avenue, Xi Zhi Men, Xicheng District, Beijing, China; 3 Ophthalmology Optometry Centre, Peking University People’s Hospital, Beijing, China; 4 Key Laboratory of Diagnosis and Therapy of Retinal and Choroid Diseases, Beijing, China

**Keywords:** Blood concentration, Estimated glomerular filtration rate, Hydroxychloroquine, Systemic lupus erythematosus

## Abstract

**Background:**

The aim of this study was to analyze the relationship of the estimated glomerular filtration rate (eGFR) to hydroxychloroquine (HCQ) blood concentrations in systemic lupus erythematosus (SLE) patients.

**Method:**

Patients with SLE who had been taking HCQ for more than 12 months were recruited. All subjects gave written informed consent. Various clinical characteristics and laboratory values were examined. The blood concentration of HCQ was measured by high-performance liquid chromatography, and the relationship of eGFR to HCQ blood concentration was mainly investigated.

**Result:**

In total, 115 patients with SLE receiving long-term HCQ therapy were included in the study. The median concentration of HCQ was 1096 ng/ml (range 116–8240 ng/ml). The eGFR was strongly associated with blood concentration of HCQ (*P* = 0.011, *P* < 0.05), when adjusted for age, sex, body mass index (BMI), weight-adjusted dose, prednisone use and immunosuppressive drug use. No statistically significant association were found between age, duration, BMI, weight-adjusted HCQ dose, corticosteroid use, immunosuppressant use and blood concentrations of HCQ.

**Conclusion:**

We provided novel evidence that impaired renal function influenced the blood concentration of HCQ. Patients with low eGFR need to adjust the HCQ dosage according to the monitoring results of HCQ blood concentrations.

**Table:** 

**Key points** *• A higher HCQ blood concentration was associated with low eGFR.* *• This finding reinforces the importance of routine HCQ measurement to maintain normal blood concentrations.* *• HCQ blood monitoring will be useful for dose modification in patients with renal dysfunction.*	

## Introduction

Hydroxychloroquine (HCQ) is a traditional antimalarial drug that is effective in the treatment of systemic lupus erythematosus (SLE). In addition to immune regulation through the inhibition of immune activation and the reduction of cytokine production, it is associated with a wide range of benefits, such as its anti-infection, anti-thrombotic, lipid-lowering, photoprotection, hypoglycemic, anti-osteoporosis, and anti-inflammation qualities, and improvement of dry eye [1, 2].

Previous studies [3–7] have identified that a very low blood concentration of HCQ is a simple marker and predictor of systemic lupus erythematosus exacerbation and treatment failure. A recent study showed that higher HCQ blood concentrations predicted retinopathy of HCQ [8]. Thus, interest in the measurement of HCQ blood concentrations has increased.

Due to its unique pharmacokinetic and pharmacogenomic characteristics, there is significant interindividual variability in HCQ blood concentrations, even if individuals take the same dose [9–11]. Several studies have analyzed the factors that influence the changes in the blood concentration of HCQ [12–15]. However, these studies have revealed contradictory findings, particularly on renal function. Most of the studies found a significant association of impaired renal function with high blood HCQ concentration [12, 14]. Another study did not find a relationship between renal function and HCQ concentration. However it should be noted that due to the small sample size of the study, only 3.7% of patients had renal dysfunction classified as CKD stage 3 or greater [13]. Another study including patients with renal dysfunction showed a trend toward lower blood HCQ concentrations [15]. However it should be noted that the patients took lower than usual doses (200 mg/day). Although the correlation between renal function and blood HCQ concentration remains controversial, we believe the negative findings could be attributed to the low power or low doses. Does the dosage of HCQ need to be adjusted in patients with renal dysfunction? How can the concentration of HCQ in the blood be modified in the presence of renal dysfunction with a decrease in eGFR? Therefore, the purpose of the study was to identify the relationship of eGFR to HCQ blood concentrations in SLE patients.

## Method

### Study design

This was a cross-sectional study aimed at exploring various factors associated with the blood concentration of HCQ, especially focusing on the effect of eGFR. The human ethics committees at the Peking University People’s Hospital approved the study (2020PHB209-01). All research adhered to the tenets of the Declaration of Helsinki. All subjects gave written informed consent.

### Population

Patients who received HCQ (400 mg/day) for at least 12 months were included in this study. Whole blood was collected, and laboratory values were collected from the electronic medical record system (HIS platform). We also analyzed blood concentrations of HCQ in patients with chronic renal insufficiency. We used the chronic kidney disease-epidemiology collaboration (CKD-EPI) equation to estimate the eGFR [16]. Renal function was classified on the basis of the stage of chronic kidney disease (CKD), with eGFR ≧90, 60–89, 30–59, 15–29, and < 15 ml/minute/1.73 m.^2^ corresponding to stage 1, 2, 3, 4, and 5 disease, respectively [17].

### Sample processing

Whole-blood HCQ can be quantified by high-performance liquid chromatography (HPLC). 300 μl of blood sample and 10 μl of 25 μl•mL^−1^ metronidazole solution which was used as the internal standard were extracted with 900 μl of ethyl acetate, redissolved with 200 μl of mobile phase after drying with nitrogen, and 20 μl of supernatant was taken for determination. Chromatographic separation was performed on a Symmetry® C_18_ column (4.6 mm x 250 mm, 5 μm) at 35℃ using a mobile phase of 20 mmol•L^−1^ KH_2_PO_4_-acetonitrile (85:15, ν/ν, pH adjusted to 3 by H_3_PO_4_) at a flow rate of 0.8 mL•min-1 and the detection wavelength was 254 nm. A calibration curve (100–5,000 ng/ml) was generated to validate the method. The relative standard deviations of intraday and interday precisions for HCQ were within 4%. The selectivity, sensitivity, precision, and accuracy of the method were established by an internal standard prior to measurement. The method was simple, sensitive, and accurate, and could be used for the measurement of HCQ blood concentrations in human blood.

### Statistical analysis

We used descriptive statistics and plots to test the data. Because the HCQ blood concentration was not normally distributed, it was natural log–transformed. Categorical variables are presented as frequencies and percentages, and continuous variables are presented as the means and standard deviations. Normally distributed values are expressed as the mean ± SD. Nonnormally distributed values were categorized into quartiles. The clinical characteristics of patients in the low and high concentration group were compared with the chi-square test for categorical variables and the Mann–Whitney test for continuous variables. The association of eGFR and HCQ blood concentration was assessed with the use of multivariable logistic regression models. According to possible confounders, we adjusted multivariable logistic regression models for age, sex, body mass index (BMI), weight-adjusted dose, and use of prednisone and immunosuppressive drugs. In another separate analysis, for multiple group comparisons distributed by CKD, one-way analysis of variance (ANOVA) or a nonparametric Mann–Whitney test was performed. The effect of eGFR on HCQ concentration was analyzed by a simple linear regression model. Statistical analyses were performed using SPSS Statistics 24.0 software (SPSS Inc., Armonk, NY, USA) and presented using GraphPad Prism 8.0 software (GraphPad Software Inc., San Diego, CA, USA). The adopted significance levels in all analyses were set at 5%.

## Result

### Clinical characteristics of patients with SLE

**Fig. 1:**
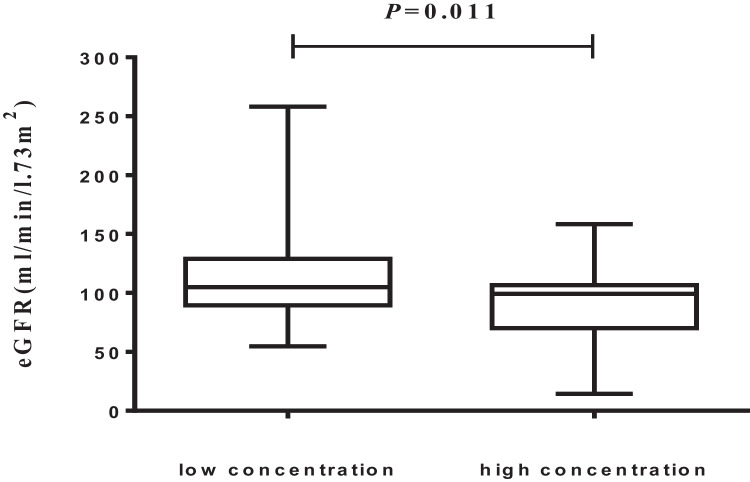
The distribution of eGFR in the low- and high-concentration groups

**Fig. 2:**
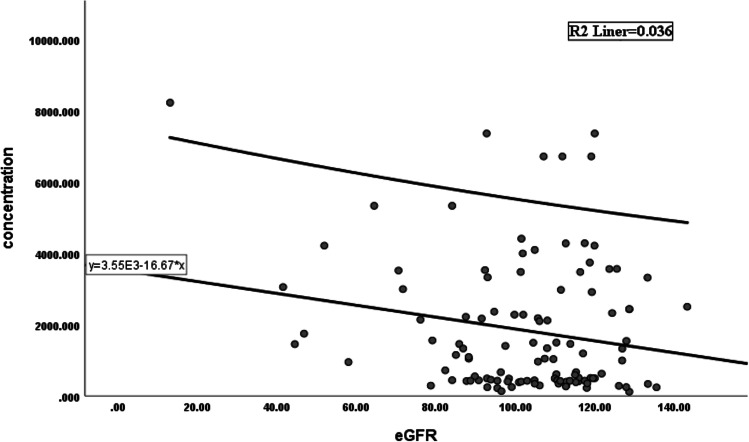
A linear regression analysis between [HCQ] concentration and eGFR

The study included 111 patients who were receiving the same daily dose of HCQ (400 mg/day every day). The median concentration of HCQ was 1096 ng/ml (range 116–8240 ng/ml). The analysis was conducted after dividing patients into two groups according to the blood concentration of HCQ. The patients with an HCQ blood concentration equal to or lower than the median (1096 ng/ml) were classified as the low concentration group (*n* = 55), and the patients with an HCQ blood concentration higher than the median (1096 ng/ml) were classified as the high concentration group (*n* = 56). The characteristics of both groups are shown in Table 1. The weight (*p* = 0.008), body mass index (*p* = 0.036), and weight-adjusted HCQ dose (*p* < 0.001) were significantly different between the two groups. The age, sex ratio, medication duration time, cumulative dose and combination therapy (prednisone and immunosuppressive drugs) were similar for the low and high blood concentration groups. We also examined the association between laboratory values and HCQ blood concentrations, and a less significant relationship was observed (Table 2). The patients with low concentrations of HCQ had a higher estimated glomerular filtration rate (eGFR) than those with high HCQ concentrations [: mean-standard deviation: (106.21 ± 14.95) vs. (99.22 ± 25.65), *p* = 0.011] (Fig. 1). By drawing the scatter plot, it is intuitively judged that there is a linear relationship between HCQ concentrations and the eGFR (F = 4.099, *P* < 0.045) (Fig. 2).

**Table 1:** Characteristics of patients in the low- and high-concentration groups

Variables	Total (*n* = 111)	Low concentration (*n* = 55)	High concentration (*n* = 56)	*P*
Gender, *n* (%)				0.716
female	104 (93.7)	51 (92.7)	53 (94.6)	
man	7 (6.3)	4 (7.3)	3 (5.4)	
Age, *n* (%)				0.957
< 60 y	84 (75.7)	41 (74.5)	43 (76.8)	
≥ 60 y	27 (24.3)	14 (25.5)	13 (23.2)	
Duration (m)	48.0 (24.0, 87.0)	48.0 (24.0, 87.0)	60.0 (24.0, 87.0)	0.786
Height (cm)	162.0 (158.0, 165.0)	162.0 (160.0, 167.0)	161.0 (155.8, 164.2)	0.148
Weight (kg)	58.0 (51.0, 66.5)	60.0 (52.5, 70.0)	55.5 (50.0, 61.5)	0.008
BMI (kg/m^2^)	22.1 (19.6, 25.1)	23.2 (20.4, 25.5)	21.2 (19.3, 24.0)	0.036
weight-adjusted dose, *n* (%)				< 0.001
≤ 5 mg/kg	28 (25.2)	28 (50.9)	0 (0)	
> 5 mg/kg	83 (74.8)	27 (49.1)	56 (100)	
cumulative dose, *n* (%)				0.716
≤ 1000 mg	80 (72.1)	41 (74.5)	39 (69.6)	
> 1000 mg	31 (27.9)	14 (25.5)	17 (30.4)	
prednisone, *n* (%)				0.176
without	36 (32.4)	14 (25.5)	22 (39.3)	
with	75 (67.6)	41 (74.5)	34 (60.7)	
immunosuppressive, *n* (%)				0.922
without	23 (20.9)	12 (22.2)	11 (19.6)	
with	87 (79.1)	42 (77.8)	45 (80.4)	
Medical history				
diabetes nephropathy	27 (24.3)	14 (51.9)	13 (48.1)	0.736
hypertensive renal disease	40 (36.0)	22 (55.0)	18 (45.0)	
diabetes nephropathy and hypertensive renal disease	5 (4.5)	2 (40.0)	3 (60.0)	
1upus nephritis	39 (35.1)	17 (43.6)	22 (56.4)	

BMI, body mass index; y, year; m, month

**Table 2:** Laboratory values of patients in the low- and high-concentration groups

Variables	Total (*n* = 111)	Low concentration (*n* = 55)	High concentration (*n* = 56)	*P*
eGFR(ml/min/1.73m^2)^	102.68 ± 21.24	106.21 ± 14.95	99.22 ± 25.65	0.011
ALB	43.01 ± 6.01	43.09 ± 5.84	42.93 ± 6.22	0.590
WBC (*10^12^/L)	5.0 (4.0, 6.7)	4.8 (4.2, 6.6)	5.2 (3.9, 6.9)	0.635
HB (g/L)	131.0 (119.5, 140.5)	132.0 (122.0, 141.0)	131.0 (114.8, 137.8)	0.336
PLT (*10^9^/L)	208.0 (171.0, 254.0)	223.0 (180.5, 265.0)	198.0 (163.8, 242.2)	0.107
ALT (U/L)	15.0 (12.0, 21.0)	15.0 (12.1, 21.0)	14.0 (11.8, 18.5)	0.603
AST (U/L)	20.0 (17.0, 23.5)	20.0 (17.0, 24.0)	19.0 (17.0, 23.0)	0.666
GGT (U/L)	17.0 (13.0, 26.0)	18.0 (13.5, 24.5)	17.0 (12.0, 26.2)	0.736
ALP (U/L)	70.0 (51.0, 88.0)	69.0 (50.0, 87.5)	70.5 (52.0, 88.0)	0.641
LDH (U/L)	179.0 (154.0, 205.0)	178.0 (155.5, 200.5)	184.5 (148.0, 208.2)	0.656
HBDH (U/L)	160.0 (136.5, 181.5)	158.0 (137.0, 181.0)	161.0 (136.2, 181.8)	0.853
CK (U/L)	64.0 (33.5, 100.5)	68.0 (39.5, 99.5)	63.5 (32.0, 102.2)	0.526
BUN (mmol/L)	4.5 (3.6, 5.3)	4.3 (3.7, 5.0)	4.7 (3.5, 5.7)	0.193
UA (μmol/L)	284.2 ± 75.8	280.3 ± 66.3	287.9 ± 84.5	0.6
TC (mmol/L)	4.4 ± 0.9	4.4 ± 0.9	4.4 ± 0.9	0.977
TG (mmol/L)	1.2 (0.9, 1.7)	1.2 (0.8, 1.7)	1.3 (1.0, 1.8)	0.401
HDL-C (mmol/L)	1.3 ± 0.4	1.3 ± 0.4	1.3 ± 0.4	0.98
LDL-C (mmol/L)	2.6 ± 0.7	2.6 ± 0.7	2.6 ± 0.6	0.913
TBIL (μmol/L)	12.0 (9.6, 14.8)	11.0 (9.2, 15.7)	12.7 (9.9, 14.3)	0.501
DBIL (μmol/L)	3.5 (2.8, 4.8)	3.4 (2.6, 4.7)	3.6 (2.9, 5.0)	0.414

eGFR, estimated glomerular filtration rate; ALB, albumin; WBC, white blood cell; HB, hemoglobin; PLT, platelet; GPT, glutamic-pyruvic transaminase; GOT, glutamic oxalacetic transaminase; GGT, gamma-glutamyltransferase; ALP, alkaline phosphatase; LDH, lactic dehydrogenase; HBDH, hydroxybutyrate dehydrogenase; CK, creatine kinase; BUN, blood urea nitrogen; UA, uric acid; TC, total cholesterol; TG, triglyceride; HDL-C, high density lipoprotein cholesterol; LDL-C, low density lipoprotein cholesterin; TBIL, total bilirubin; DBIL, direct bilirubin

### The relationship of renal function to blood concentrations of HCQ

Through multivariate analysis, we identified independent factors related to high blood concentrations of HCQ. The eGFR was independently associated with a high blood concentration of HCQ. There was a significant relationship between the eGFR and blood HCQ concentration in unadjusted models (*p* = 0.006) and in Model 1, which was adjusted for age, sex, BMI, weight-adjusted dose, use of prednisone and immunosuppressive drugs (*p* = 0.005) (Table 3).

**Table 3:** Multivariable logistic regression models evaluating the association between renal function and HCQ blood concentration

Variable	No.(%) of study participants		OR (95% CI)		*P* value	
	Low concentration	High concentration	Crude	Adjusted*	Crude	Adjusted*
eGFR	55(49.5)	56 (50.5)	0.98 (0.97 ~ 0.99)	0.97 (0.95 ~ 0.99)	0.006	0.005

*Adjust for age, sex, BMI, weight-adjusted dose, use of prednisone and immunosuppressive drugs

When patients were categorized according to CKD stage, only 6 patients had chronic renal insufficiency (eGFR < 60 ml/min); among them, five people had CKD stage 3, and one person had CKD stage 4. These patients also took the same daily dose of HCQ (400 mg/day every day), and it was not adjusted to take CKD into account. The median blood concentration of HCQ was 2404.04 ng/ml (950.80–8240.20 ng/ml) and was significantly higher than the median blood concentration of HCQ in the 105 patients of the study who also received 400 mg/day (1046 ng/ml [range 116–7374.89 ng/ml]; *P* = 0.049). There was no significant difference in HCQ blood concentration according to the five different CKD stages, but there was a trend towards a difference but, perhaps due to the small sample size, the difference was not significant. When one patient had CKD stage 4, the HCQ blood concentration was especially high (*n* = 1, 8240.0 ng/mL) (Table 4).

**Table 4:** HCQ blood concentrations according to CKD stage

CKD stage	No.(%)	Blood HCQ concentration	Crude		Adjusted	
			OR (95% CI)	*P* value	OR (95% CI)	*P* value
CKD 1	87	963.71 (407.39, 2505.65)	(Ref)		(Ref)	
CKD 2	18	1242.02 (521.97,2416.97)	0.13 (-0.05 ~ 0.32)	0.168	0.15 (-0.05 ~ 0.35)	0.152
CKD 3	5	1749.40 (1202.75,3640.67)	0.32 (-0.09 ~ 0.74)	0.124	0.41 (-0.06 ~ 0.87)	0.088
CKD 4	1	8240.0 (8240.0, 8240.0)	0.94 (0.05 ~ 1.84)	0.041	0.84 (-0.04 ~ 1.73)	0.065
Trend	111		0.18 (0.04 ~ 0.31)	0.012	0.2 (0.04 ~ 0.35)	0.013

*Adjust for age, sex, BMI

## Discussion

In this study, we identified factors that might explain interindividual variations in blood concentrations of HCQ in patients with SLE. Interestingly, we identified a clear correlation between eGFR and blood concentration of HCQ. Although several previous studies [12–15] examined the relationship between HCQ blood concentrations and variables such as age, BMI, smoking, drug-drug interactions, dosage, laboratory examination (white blood cell, blood platelet, hemoglobin, neutrophil, etc.), few have studied renal function. We found a significant association of low eGFR with high blood concentrations of HCQ.

Similarly, Ji Yeon Lee et al. conducted a cross-sectional study to explore the relationship between renal function and blood concentration of HCQ. They found high blood HCQ concentrations in 4 patients with abnormal eGFR compared with 23 SLE patients with normal eGFR [13]. Another study performed by M. Jallouli et al. also found an inverse correlation between the estimated glomerular filtration rate and HCQ blood concentration, and in their study, they also studied three patients receiving long-term dialysis and confirmed that HCQ was not dialyzable [12].

However, a study including 15 patients with renal dysfunction (creatinine: 1.4–4.9 mg/ml) and 6 patients with more severe renal dysfunction (creatinine > 5.0 mg/ml) showed a trend toward lower HCQ concentrations with renal failure, suggesting that renal failure dosing led to suboptimum HCQ concentrations [15]. Although our study had an opposite conclusion to the above two studies, we thought the reason for the inconsistency was the lower than usual doses, in which the patients only received HCQ doses of 200 mg/d. On the other hand, it suggested that blindly reducing the dose was not the best way to quantitate renal disease with respect to HCQ dosing. Moreover, another study also found no statistically significant association between renal function and [HCQ] or [DHCQ]. However, the author recognized that the study population was not ideal for studying the relationship due to low power [14]. Although it is still controversial to conclude that HCQ blood concentrations are associated with renal function, our positive finding could be attributed to high power. As previously described, retinal toxicity [18, 19], neuromyotoxicity [20] and cardiotoxicity [21] of HCQ may be enhanced by renal dysfunction. HCQ can lead to retinal toxicity, and an increasing number of patients with advanced HCQ retinopathy have been had identified in recent publications, thus suggesting a need for guidelines that focus on recommending appropriate dosing and toxicity monitoring [22, 23]. The most important risk factor is greatly dependent on daily dose, which is calculated using body weight. Guidelines aimed at preventing retinal toxicity recommend using less than 5 mg/kg/day of actual body weight per day instead of 6.5 mg/kg/day of ideal body weight. To date, there are few guidelines for dose adjustment in patients with renal dysfunction. The Joint European League Against Rheumatism and European Renal Association–European Dialysis and Transplant Association (EULAR/ERA–EDTA) guidelines [22] state that HCQ is recommended for all patients without contraindications, with a maximum dose of 5 mg/kg/day. When GFR < 30 ml/min, the dose can be reduced by 50%. Kidney Disease: Improving Global Outcomes (KDIGO) guidelines [23] also state that HCQ is appropriate for all patients without contraindications, but the recommended dosage varies. Guidelines recommend an initial dose of 6.5 mg/kg/day of ideal body weight or 400 mg/ day, and 4–5 mg/kg/day during maintenance treatment. A dose reduction of at least 25% is recommended when eGFR is less than 30 ml/min/1.73m^2^. We think that HCQ blood monitoring will be useful. Therefore, further studies are needed in patients with renal dysfunction to confirm our findings and to examine the association among renal function, HCQ blood concentration and toxicity.

## Conclusion

In conclusion, we provide novel evidence that a higher HCQ blood concentrations are associated with low eGFR. This finding reinforces the importance of routine HCQ measurement to maintain normal blood concentrations. HCQ blood monitoring will be useful for dose modification in patients with renal dysfunction. With the popularization of blood drug concentration determination, such data might be useful for clinicians.

## Data Availability

The original contributions presented in the study are included in the article, and further inquiries can be directed to the corresponding author.
